# Evidence for Beneficial Associations between Isoenergetic Macronutrient Exchanges and Serum non-HDL Cholesterol, a Measure of All Circulating Atherogenic, apoB-Containing Lipoproteins

**DOI:** 10.1093/jn/nxab127

**Published:** 2021-05-12

**Authors:** Bruce A Griffin, Julie A Lovegrove

**Affiliations:** Department of Nutritional Sciences, Faculty of Health and Medical Sciences, University of Surrey, Guildford, Surrey, United Kingdom; Hugh Sinclair Unit of Human Nutrition, Department of Food and Nutritional Sciences, University of Reading, Reading, Berkshire, United Kingdom; Institute for Cardiovascular and Metabolic Research, Department of Food and Nutritional Sciences, University of Reading, Reading, Berkshire, United Kingdom

See corresponding article on page 2317.

The replacement of SFAs with unsaturated fatty acids remains a cornerstone of current dietary guidelines to reduce risk of atherosclerotic cardiovascular disease (ASCVD) (1). Although much of the basis for this specific guideline rests on the lowering of serum LDL cholesterol, there is evidence to suggest that this dietary exchange may have an impact on other cardiometabolic risk factors (2, 3). Low serum HDL cholesterol (<40 mg/dL) and elevated non-HDL cholesterol (>130 mg/dL) are relevant in this respect, the latter because it represents a measure of cholesterol in all circulating atherogenic, apoB-containing lipoproteins [VLDL, IDL, LDL, Lipoprotein (a), and lipoprotein remnants] (4). The response of serum LDL cholesterol to isoenergetic macronutrient exchanges has been well documented in meta-analyses and shown to occur in a dose-response fashion (5, 6); there is, however, less evidence for the effects of these dietary exchanges on serum non-HDL cholesterol.

The study by Pinart et al. (7) in this issue of *The Journal of Nutrition* reports a study-level meta-analysis (or federated meta-analysis) in which the analysis was undertaken in each of the included studies separately, followed by a combination of all estimates and SEs using conventional study-level meta-analysis. The dietary exchange model involved the isoenergetic replacement of 5% of total energy from total carbohydrates with either total fat, SFAs, PUFAs, or MUFAs. Associations between these dietary exchanges and serum HDL and non-HDL cholesterol were examined in 5919 male and female (54%) participants, aged between 15 and 65 y, from 8 observational European studies. Five of these studies were longitudinal and 3 cross-sectional, in which data from a baseline or single follow-up visit were used in the dietary exchange model. The studies, which were predominantly German (5 of 8) with others from Belgium, Italy, and Spain, were part of the European Nutritional Phenotype Assessment and Data Sharing Initiative. Dietary intake was assessed by a variety of validated, self-completed methods that were specific to each country. Data were examined primarily by virtual individual person data analysis, and a study-level meta-analysis general linear model to compare results in a secondary analysis.

In models adjusted for sex, age, smoking, and BMI, the replacement of carbohydrate with SFAs or PUFAs was shown to be unrelated to fasted HDL cholesterol, but associated with a higher and lower non-HDL cholesterol, respectively (SFAs: 1.94 mg/dL; 95% CI: 0.08, 3.79 mg/dL; *P* = 0.04; and PUFAs: −3.91 mg/dL; 95% CI: −6.98, −0.84 mg/dL; *P* = 0.01). In contrast, replacement of carbohydrate with either total fat or MUFAs was associated with a higher HDL cholesterol (total fat: 0.67 mg/dL; 95% CI: 0.40, 0.94 mg/dL; *P* < 0.0001; and MUFAs: 0.99 mg/dL; 95% CI: 0.37, 1.60 mg/dL; *P* = 0.002), but was unrelated to non-HDL cholesterol. Age was unrelated to these associations, and there was an inverse association between non-HDL cholesterol in men and the replacement of carbohydrate with MUFAs, although substantial heterogeneity in the data presumably precluded subgroup analysis. The replacement of 5% of energy from carbohydrates with total fats was more strongly associated with higher HDL cholesterol in women (0.84 mg/dL; 95% CI: 0.46, 1.21 mg/dL) than in men (0.44 mg/dL; 95% CI: 0.07, 0.82 mg/dL; *P*-interaction = 0.05).

The positive and potentially favorable associations between the replacement of carbohydrate with total fat or MUFAs and serum HDL cholesterol are consistent with the established effects of these dietary exchanges from meta-analyses of randomized controlled trials (RCTs) (5, 6). The lack of association between replacing carbohydrate with total fat and non-HDL cholesterol could suggest that greater emphasis should be placed on fat quality, rather than quantity, for reducing cardiometabolic risk. A less consistent result with previous findings was the absence of significant associations between carbohydrate replacement with PUFAs or SFAs and HDL cholesterol. In meta-analyses of RCTs, both LDL and HDL cholesterol have been reported to show positive associations with the addition or removal of dietary PUFAs, MUFAs, and SFAs with a chain length of 12–16 carbons (5, 6). The lack of evidence for these associations in this study may reflect a relative weakness in the modeling of observational data as compared with that from intervention trials.

The interpretation of what may seem like a counterintuitive positive association between serum HDL cholesterol and dietary SFA in meta-analyses of RCTs requires consideration of how dietary SFA influences the structure and functional properties of this antiatherogenic lipoprotein. In this respect, associations between macronutrient exchanges and serum HDL cholesterol may be of less value in understanding the link between diet, HDL particles, and ASCVD, in much the same way as the cholesterol content of LDL particles conveys less information about LDL atherogenicity than the number and average size of LDL particles (8). Emerging evidence from studies on the impact of macronutrient composition on the functional properties of HDL is beginning to shed light on mechanistic links with ASCVD (9).

Another point to consider is whether the magnitude of these statistically significant associations, in terms of higher and lower non-HDL cholesterol, is of clinical significance to ASCVD? An increase in non-HDL cholesterol of 1 mg/dL has been reported to be associated with a 5% increased risk of coronary artery disease death in men and women with diabetes, whereas the same change in LDL cholesterol was associated with a lower, 4% increased risk of coronary artery disease death (10). Although diabetes is a disease with a high risk of ASCVD, the higher and lower concentrations of non-HDL cholesterol associated with the replacement of carbohydrate with SFAs (+1.94 mg/dL) and PUFAs (−3.91 mg/dL), respectively, give credibility to the clinical significance of these values.

The study design used carbohydrate as the reference macronutrient in its isoenergetic exchange models. It also claimed that the main findings were reproduced when protein instead of carbohydrate was replaced with dietary fats (data not shown). Although it is possible to interpret the outcome of these dietary exchanges in terms of what might happen when fat is replaced with different fats or carbohydrate, the isoenergetic replacement of SFAs with PUFAs and/or MUFAs would have provided more direct evidence to support the potential benefit of current dietary guidelines to replace SFAs with unsaturated fats. Moreover, the inclusion of data on serum LDL cholesterol would have allowed a direct comparison of the relative efficacy of these dietary exchanges on serum LDL and non-HDL cholesterol. It would also have been possible to estimate remnant lipoprotein cholesterol (total cholesterol minus HDL cholesterol minus LDL cholesterol) as an important atherogenic component of non-HDL cholesterol (11).

Rigor in the choice and application of statistical methods, and the harmonization of data across centers, were listed as major strengths in the present study—in particular, the use of a remote federated analysis (DataSHIELD), which allowed both study-level and virtual individual person data meta-analyses, without the need to pool or share individual‐level data. This approach was reported to offer some advantages by reducing governance burdens and ethico-legal challenges.

Limitations of the study include the inevitable drawback of cross-sectional studies producing evidence for associations between macronutrients and serum lipids, rather than causality. Outcomes were also subject to residual confounding, and in coming from just 4 countries may not be representative of the diverse European population. Perhaps more critically, the quality and food sources of macronutrients were not assessed in this study and are known to exert differential effects on blood lipids and cardiovascular disease risk (12). This includes the quality of carbohydrates, e.g., whole grains compared with simple carbohydrates (13), and dietary fats, e.g., n–6 and n–3 PUFAs (14). It also applies to the differential effects of MUFAs from plants and animals (15), and SFAs from dairy foods and other animal sources (16).

Notwithstanding these and other methodological limitations, the study by Pinart et al. (7) provides indirect evidence to support the benefits of replacing dietary SFAs with unsaturated fats, on serum lipids with greater relevance to cardiometabolic risk than LDL cholesterol.

## Notes

The authors reported no funding received for this study.

Author disclosures: JAL is Deputy Chair of the UK Government's Scientific Advisory Committee on Nutrition (SACN) and was a member of the SACN's Working Group on “Saturated Fats and Health.” JAL chairs the International Life Sciences Institute committee on “Individual Saturated fatty acids and Cardiovascular Risk.” BAG reports no conflicts of interest.
